# Rational identification of aggregation hotspots based on secondary structure and amino acid hydrophobicity

**DOI:** 10.1038/s41598-017-09749-2

**Published:** 2017-08-25

**Authors:** Daisuke Matsui, Shogo Nakano, Mohammad Dadashipour, Yasuhisa Asano

**Affiliations:** 1 Biotechnology Research Center and Department of Biotechnology, Toyama Prefectural University, 5180 Kurokawa, Imizu Toyama, 939-0398 Japan; 2 Asano Active Enzyme Molecule Project, ERATO, JST, 5180 Kurokawa, Imizu Toyama, 939-0398 Japan; 3 Graduate School of Pharmaceutical and Nutritional Sciences, University of Shizuoka, 52-1 Yada, Suruga-ku Shizuoka, 422-8526 Japan

## Abstract

Insolubility of proteins expressed in the *Escherichia coli* expression system hinders the progress of both basic and applied research. Insoluble proteins contain residues that decrease their solubility (aggregation hotspots). Mutating these hotspots to optimal amino acids is expected to improve protein solubility. To date, however, the identification of these hotspots has proven difficult. In this study, using a combination of approaches involving directed evolution and primary sequence analysis, we found two rules to help inductively identify hotspots: the α-helix rule, which focuses on the hydrophobicity of amino acids in the α-helix structure, and the hydropathy contradiction rule, which focuses on the difference in hydrophobicity relative to the corresponding amino acid in the consensus protein. By properly applying these two rules, we succeeded in improving the probability that expressed proteins would be soluble. Our methods should facilitate research on various insoluble proteins that were previously difficult to study due to their low solubility.

## Introduction

Enzymes catalyze various reactions that are difficult to achieve by current technologies for chemical catalysis. Advances in molecular biology enable us to use various recombinant proteins and enzymes to synthesize fine chemical compounds, such as medicine precursors and pesticides. Currently, recombinant proteins can be obtained using heterologous expression systems^1^. In these systems, prokaryotes such as *Escherichia coli* are widely used because they are easy to handle and produce high levels of proteins^2^. Despite these advantages, the *E. coli* expression system also has several drawbacks. In particular, the formation of inclusion bodies (IBs) often decreases the yield of target protein in the soluble fraction^3^. For example, approximately 85% of the cloned open reading frames (ORFs) from *Caenorhabditis elegans* are expressed in insoluble fractions^4^. Therefore, new methods are required to reduce the formation of IBs and improve the solubility of target proteins in the *E. coli* expression system. In general, protein solubility can be classified into the following two ways: *in vitro* solubility, which is affected by the refolding efficacy of the proteins from IBs, and *in vivo* solubility, which is affected by the efficacy of soluble expression in the *E. coli* cytoplasm^5^.

Various methods have been reported to increase the yield of solubilized recombinant proteins. The simplest and most widely applied method is the optimization of the culture conditions of the strain, e.g., lowering the cultivation temperature^6^ and adding metal ions or precursors of cofactors to the cultures^7^. *E. coli* strains that express high levels of chaperones, such as DnaK or GroEL, are also effective for improving solubility^8, 9^; in this case, the chaperone assists with *in vivo* refolding of the proteins. When such methods cannot improve the solubility, however, an alternative method is direct modification of the sequence of the target protein (STP)^3^. For such modifications to be successful, it is first necessary to identify aggregation hotspots, i.e., amino acid residues that affect the formation of IBs in the target protein^2, 3^. After being identified, the hotspot residues are mutated to other amino acids to improve the solubility. However, identification remains difficult because various factors such as the size of polypeptides, total protein charge, and phylogenetic origin affect the solubility of target proteins^3, 10^. Computational methods such as SOLpro^11^ and PROSO II^12^ have been developed to estimate whether a given target protein would be soluble in a heterologous expression system. PROSS can optimize the sequence of a target protein to improve protein stability and solubility in a heterologous expression system by introducing dozens of mutations simultaneously, based on the results of multiple sequence alignment (MSA) and structural data^13, 14^. In addition, hotspots can be predicted using software such as AGGRESCAN^15^, SolubiS^16^, and CamSol^17^.

For such difficult cases, learning from successful examples in previous research would facilitate development of a new method of estimation. In a previous study, we discovered a phenomenon involving the soluble expression of a plant enzyme, hydroxynitrile lyase from *Manihot esculenta* (*Me*HNL), in *E. coli*. When the enzyme was subjected to random mutagenesis, the single point mutation H103L and simultaneous mutation of three surface residues (Lys to Pro) conferred total solubility in *E. coli*, even when the cells were grown at 37 °C, facilitating the industrial use of *Me*HNL^18^. Prompted by this study, we began to accumulate more examples of enzymes made soluble through mutagenesis. Based on these examples, we characterized the relationship between enzyme structures and solubility in heterologous hosts such as *E. coli*, using *Me*HNL as a model, and extended the concept to screen and classify several enzymes/proteins and mutate them as much as possible to achieve soluble expression in *E. coli*. Thus, through random mutagenesis of insoluble proteins, it will become possible to elucidate the relationship between correct folding in *E. coli* and primary and higher-order structures. Following organization of the results, this approach will enable an analysis of the relationship primary structure and protein expression. Thus, we were able to find rules using an inductive method. If such rules could be established, it would become easier to generate a mutant that could be expressed and correctly folded in *E. coli*. It would then in turn become much easier to express enzymes/proteins in heterologous hosts, avoiding the current trial-and-error methods. Hence, we developed a new method to predict hotspots without a crystal structure, with the goal of solubilizing expressed proteins.

First, we solubilized four insoluble proteins by directed evolution methods using the *E. coli* expression system: mandelonitrile oxidase from the millipede *Chamberlinius hualienensis* (*Ch*MOX; GenBank accession number LC036560), l-arginine decarboxylase from the plant *Arabidopsis thaliana* (*At*ADC; GenBank accession number NP_179243.1), and l-glutamate dehydrogenase (*Dm*GDH; GenBank accession number NP_651140.1) and l-ornithine decarboxylase (*Dm*ODC; GenBank accession number CAA47165.1) from the insect *Drosophila melanogaster*. Similar results were obtained through mutation experiments, the secondary structure analysis, and hydropathy analysis. Taking advantage of the differing perspectives of sequence conservation and hydropathy contradiction, we then developed a method to assign the hotspots using INTMSAlign, a program for identifying consensus residues^19^. The prediction of hotspots in the target proteins, such as l-phenylalanine dehydrogenase from the bacterium *Sporosarcina ureae* (*Su*PDH; GenBank accession number BAA19221.1), was successfully achieved in regard to the HiSol score, which is derived from the secondary rule and calculated using the output file of INTMSAlign and the hydropathy index. Furthermore, using the methods we developed, we solubilized luciferase from the crustacean *Metridia pacifica* (*Mp*LUC; GenBank accession number LC155420) by mutating multiple residues. Our findings demonstrate that this approach represents an alternative method, developed inductively from analysis of results described here, for predicting aggregation hotspots.

## Materials and Methods

### Materials

A complementary DNA (cDNA) library of *A. thaliana* was purchased from Life Technologies (Carlsbad, CA, USA). Poly (A)^+^ RNAs of *D. melanogaster* were purchased from CLONTECH Laboratories, Inc. (Palo Alto, CA, USA). PrimeSTAR GXL DNA polymerase, cold-shock expression vector pColdI, Tks Gflex DNA polymerase, restriction enzymes, and In-Fusion HD Cloning Kit were purchased from TaKaRa Bio (Shiga, Japan). The prokaryotic expression system (pET-11a and pET-22b) and BugBuster reagent were purchased from Novagen (Darmstadt, Germany), coelenterazine and *E. coli* BL21 (DE3) from Promega (WI, USA), and *E*. *coli* XL-1 Red and QuikChange Lightning (Multi) Site-Directed Mutagenesis Kit from Stratagene (La Jolla, CA, USA). l-Amino acids were purchased from Peptide Institute, Inc. (Osaka, Japan), and (*R*)-mandelonitrile from Sigma-Aldrich Co. (St. Louis, MO, USA). All other chemicals were purchased from Kanto Kagaku Co. (Tokyo, Japan), Nacalai Tesque, Inc. (Kyoto, Japan), or Tokyo Kasei Kogyo (Tokyo, Japan), unless otherwise stated, and were of the highest grade commercially available.

### Construction of expression plasmids and growth conditions

The previously constructed plasmids pET-22b-*chmox* and pUC-18-*supdh*, were used for expression of the *Ch*MOX gene (*chmox*) and the *Su*PDH gene (*supdh*), respectively^20, 21^. The cDNA of *Mp*LUC was synthesized and amplified using *PfuUltra* II fusion HS DNA polymerase (Agilent Technologies, Santa Clara, CA, USA) and primers P1 and P2 (Table S1). After digestion with *Nde*I and *Xho*I, each amplified gene was ligated into pColdI with T4 DNA ligase (TaKaRa Bio). The *A*. *thaliana* cDNA library was used as a template for polymerase chain reaction (PCR). The cDNA (*atadc*) encoding arginine decarboxylase (*At*ADC) was amplified using Tks Gflex DNA polymerase and primers P3 and P4 (Table S1). After digestion of pET-11a with *Nde*I and *Bam*HI, each amplified gene was ligated into pET-11a using In-Fusion HD Cloning Kit, generating pET-11a-*atadc*. Poly (A)^+^ RNAs of *D. melanogaster* were used to generate cDNA by reverse transcription using the Prime Script RT reagent kit. The cDNAs encoding *Dm*GDH and *Dm*ODC were amplified using PrimeSTAR GXL DNA polymerase and primers P5–P8 (Table S1). After digestion of pET-11a with *Nde*I and *Bam*HI, each amplified gene was ligated into pET-11a using the In-Fusion HD Cloning Kit to generate pET-11a-*dmgdh* and pET-11a-*dmodc*.

Plasmids pET-22b-*chmox*, pET-11a-*atadc*, pET-11a-*dmgdh*, pET-11a-*dmodc*, and pColdI-*mpluc* were transformed into *E*. *coli* BL21 (DE3). The transformants were grown in Luria–Bertani (LB) medium consisting of 1.0% peptone, 1.0% NaCl, and 0.5% yeast extract containing 100 μg/ml ampicillin at 37 °C in a stroke shaker at 300 rpm until the optical density at 600 nm reached 0.5–0.6. Protein expression was induced at 16 °C for 16 h or at 30 °C for 6 h by addition of 0.5 mM (final concentration) isopropyl-β-d-1-thiogalactopyranoside (IPTG). Plasmid pUC18-*supdh* was transformed into *E*. *coli* JM109, and the transformants were grown at 37 °C for 17 h in LB medium containing 100 μg/ml ampicillin and 1.0 mM IPTG. The expression of each soluble or insoluble fraction was detected by sodium dodecyl sulfate polyacrylamide gel electrophoresis (SDS-PAGE) using the Laemmli method^22^. Each soluble protein was purified by nickel-immobilized metal affinity chromatography (His GraviTrap, GE Healthcare, Waukesha, WI, USA).

### Assays of enzyme activities

*Ch*MOX activity towards (*R*)-mandelonitrile was assayed by the color development method^21^. Activities of *At*ADC towards l-arginine and *Dm*ODC towards l-ornithine were assayed by the color development method, coupled with putrescine oxidase from *Micrococcus rubens* IFO 3768^23^. Activities of *Dm*GDH towards l-glutamate and *Su*PDH towards l-phenylalanine were measured by monitoring the production of β-NADH at 340 nm^24^. *Mp*LUC activity was measured according to the luminescence spectra method^25^. Protein concentrations were determined using the Bradford protein assay^26^, using a dye reagent concentrate (Bio-Rad, Richmond, CA, USA) with bovine serum albumin as the standard.

### Screening of soluble and active variants

*E*. *coli* XL-1 Red was used for random mutagenesis of pET-22a-*chmox*, pET-11a-*atadc*, pET-11a-*dmgdh*, and pET-11a-*dmodc* as previously described^27^. *E*. *coli* BL21 (DE3) was transformed with the resultant plasmids, and the transformants were incubated on LB agar plates containing 100 μg/ml ampicillin. Colonies were picked from the agar plates using a QPix 420 Bench-top Colony Picker (Molecular Devices, CA, USA) and placed into 96-well plates with 300 μl of LB medium containing 50 μg/ml ampicillin. The cells were incubated as described in “Construction of expression plasmids and growth conditions.” After collection of the cells by centrifugation at 3,220 × *g* for 15 min, the cell pellets were disrupted with 50 μl of BugBuster reagent. The cell debris was removed by centrifugation at 3,220 × *g* for 15 min, and the supernatant was used for the activity assay. DNA sequences were analyzed on an ABI PRISM 310 genetic analyzer (PE Applied Biosystems, Carlsbad, CA, USA).

### Structure predictions and helical wheel depiction

Secondary structures were predicted using the program PSIPRED^28^ (http://www.psipred.net). The 3D structural models were predicted using SWISS-MODEL^29^ (http://swissmodel.expasy.org/). For helical wheel depiction, the EMBOSS pepwheel software^30^ (http://emboss.sourceforge.net/apps/release/4.0/emboss/apps/pepwheel.htm) was used.

### Determination of expression levels using Western blotting

The amounts of soluble and total (soluble plus insoluble) expressed *Ch*MOX wild type (WT) and variants were assayed by Western blotting. Each sample was prepared and electrophoresed as previously described^31^. Proteins on polyvinylidene fluoride membranes were visualized using anti-His tag mAb HRP DirectT (1/5,000, MBL Co., Ltd., Nagoya, Japan). Immunoblot signals were detected using the enhanced chemiluminescence Western Blotting Detection Reagents (GE Healthcare).

### Creation of site-directed mutants of *Ch*MOX, *At*ADC, *Dm*GDH, *Dm*ODC, *Su*PDH, and *Mp*LUC

Saturation site-directed mutagenesis at Val444 and Val455 of *Ch*MOX and Leu435 and Lys441 of *At*ADC was performed using oligonucleotide primers P9–P16 (Table S1). The target amino acid positions (Val444, Val455, Leu435, and Lys441) were coded by NNS. Amino-acid substitutions in *Su*PDH and *Mp*LUC were introduced using oligonucleotide primers P17–21 (Lys148 to Ile, Val or Leu; Gln225 to Val; Gln243 to Val or Ala; Gln337 to Ile; and Lys374 to Ile or Val in *Su*PDH; Table S1) and P21–P25 (Ile80 to Lys and Ala177 to Asp in *Mp*LUC; Table S1), respectively. The reactions were performed using the QuikChange Lightning (Multi) Site-Directed Mutagenesis Kit. The mutated plasmids were transformed into *E*. *coli* BL21 (DE3) or JM109, and the transformants were incubated overnight in 5 mL of LB medium containing suitable antibiotics. The randomized plasmid library was isolated and subjected to DNA sequencing, and mutations were confirmed using an ABI PRISM 310 genetic analyzer. We declare that live vertebrates were not used as samples in this study.

### Development of the software INTMSAlign_HiSol to assign aggregation “hotspots”

Utilizing the sequence of the target protein (STP), residues with hydrophobic contradiction were identified based on our defined HiSol score as follows:

1 $${\rm{HiSol}}\,{\rm{score}}({\rm{j}})={({I}_{hyp})}_{j,STP(i)}-\sum _{i=1}^{20}{({I}_{hyp})}_{i}\{{({R}_{cons})}_{ij}/100.0\}$$

The score was calculated using the output file of INTMSAlign and the hydropathy index, which was normalized to an average of “0” and variance of “1”. The score was calculated for each residue of the STP. *I*_hyp_ is the normalized hydropathy index^32^. In (*I*_*hyp*_)_*j*_,_*STP*(*i*)_, the variable “*i*” represents the amino acid residues numbered in alphabetical order of their one-letter abbreviations; for example, the *i* values of Ala, Cys and Asp are 1, 2, and 3, respectively. Thus, (*I*_*hyp*_)_*j*, STP(*i*)_ refers to the hydropathy index of the amino acid residue “*i*” at the *j*th residue in the STP. (*R*_*cons*_)_ij_ is derived from the output file of INTMSAlign; this parameter represents the appearance rate of that amino acid at the *j* ^th^ residue of a target protein in the library. A function to calculate the HiSol score was implemented in the graphical user interface of INTMSAlign^19^. In this study, we regarded residues with high absolute HiSol scores as aggregation hotspots.

The locations of the identified hotspots were classified according to the secondary structure of STP, which was estimated by PSIPRED. To summarize the identification of hotspots by the HiSol score and classification of the spots based on secondary structure, we developed a derivative of INTMSAlign, INTMSAlign_HiSol. This program requires no 3D structural information to estimate hotspots.

## Results

### Rule 1: Residues identified as aggregation hotspots based on hydropathy mismatch in α-helix structure affect protein solubility

First, we tried to find a rule for improving solubility of insoluble proteins by a combination of random mutagenesis and enzyme activity measurement. For this effort, we used the following four insoluble enzymes: *Ch*MOX, *At*ADC, *Dm*GDH, and *Dm*ODC. Libraries of 12,000 clones of each of these four enzymes were obtained by random mutagenesis, and their enzyme activities were determined by the color development method^21, 23^ to obtain soluble forms with enzyme activity. Several mutants with activity were obtained from each of the four enzymes, and the following amino acid substitutions were identified: V455D (GTT → GAT) in *Ch*MOX, K441L (AAG→TTG) in *At*ADC, V174D (GTG→GAT) in *Dm*GDH, and K117L (AAG→TTG) in *Dm*ODC. We then analyzed their amino acid sequences and confirmed that codons rare in *E. coli* were not present in any of the obtained mutants^33^ (Fig. S1A,C,E,G). Therefore, we presumed that the amino acid substitutions affected protein folding in *E*. *coli* BL21 (DE3).

**Figure 1:**
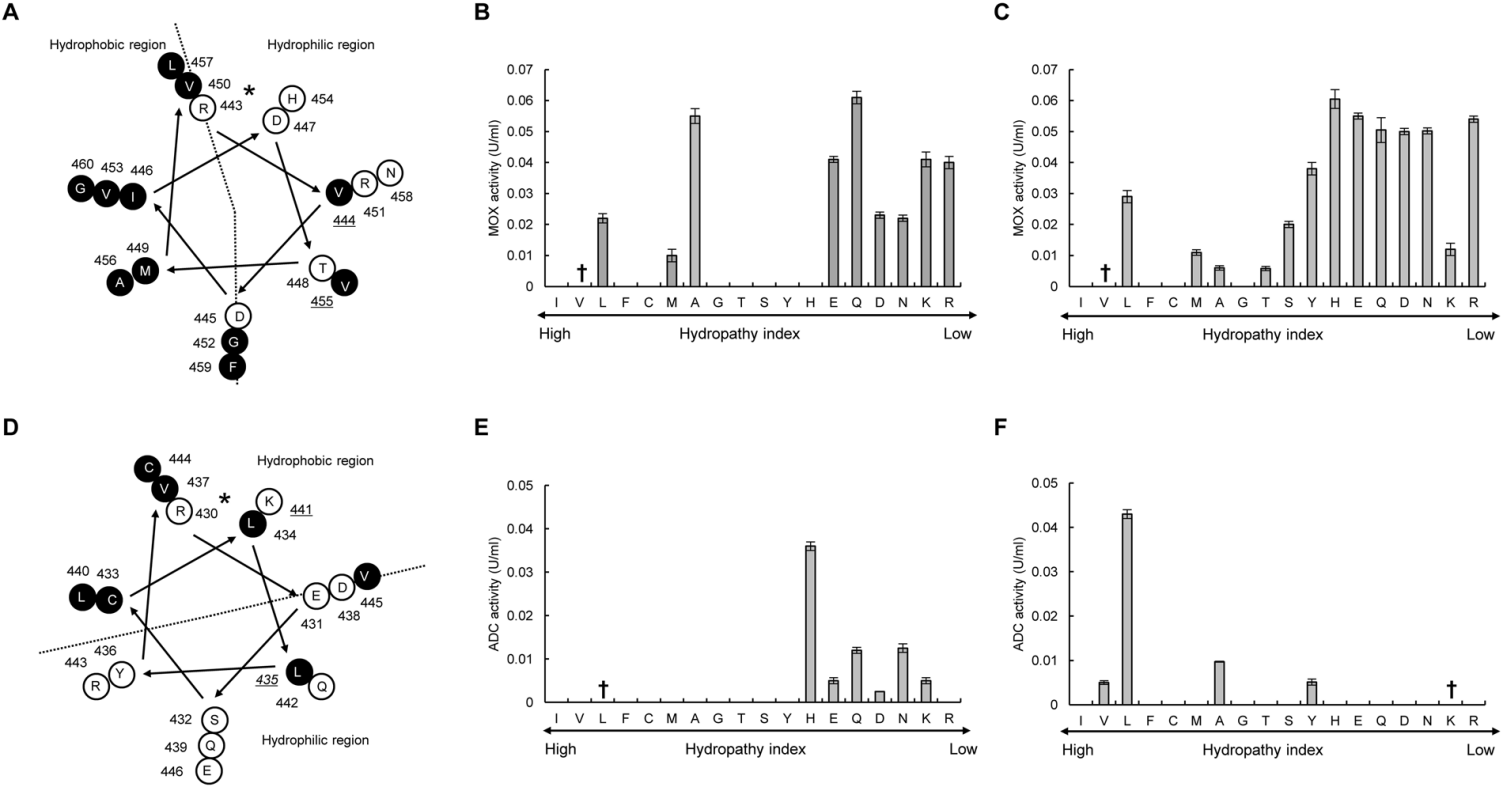
Helical wheel depictions for α-helix regions of four enzymes that contributed to improving the protein solubility, and saturation mutagenesis at aggregation hotspots on α-helices of *Ch*MOX and *At*ADC identified by directed evolution analysis. Helical wheels depict the following α-helix regions: residues 443–460 (RVDIDTMVRGVHVALNFG) of *Ch*MOX (**A**) and residues 430–446 (RESCLLYVDQLKQRCVE) of *At*ADC (**D**). Hydrophobic and hydrophilic residues are shown in white and black letters, respectively. The mutation sites for saturation mutagenesis are represented by underlined residue numbers. An asterisk (*) adjacent to the sequence number indicates the first residue of an α-helix. The enzyme activity of the saturated mutants was measured for the following four residues: V455X (**B**) and V444X (**C**) for *Ch*MOX and K441X (**E**) and L435X (**F**) for *At*ADC. The residues with low and high hydropathy indices are hydrophilic and hydrophobic, respectively. The dagger (†) indicates WT *Ch*MOX (**B**,**C**) and *At*ADC (**E**,**F**). Trp and Pro are not shown in these figures because the side chains of these residues exhibit hydrophobic character despite being classified as hydrophilic groups in the hydropathy index.

Next, we analyzed the secondary structures of the obtained mutants. Homology modeling of the enzyme structures revealed that the four mutated residues were all located in α-helix structures and exposed to the solvent (Fig. S1B,D,F,H,J). In general, hydrophobic amino acids are directed towards the interior of the protein (hydrophobic core), and hydrophilic (polar) amino acids are exposed to the solvent (surface of the protein)^34^. We further analyzed the position of the mutated residues in the α-helix structure using the helical wheel depiction. The hydrophobic residues Val455 in *Ch*MOX (Fig. 1A) and Val174 in *Dm*GDH were located in hydrophilic regions of the α-helix structure. On the other hand, the hydrophilic residues Lys441 in *At*ADC (Fig. 1D) and Lys117 in *Dm*ODC were located in hydrophobic regions of the same secondary structure.

These results indicated that mutation of hydrophobic amino acids to hydrophilic amino acids in the hydrophilic regions of the α-helix structure, or of hydrophilic amino acids to hydrophobic amino acids in the hydrophobic regions of the α-helix structure, might affect soluble expression. Kamtekar *et al*. reported that proteins could be designed based on the binary patterning of polar and nonpolar amino acids in the α-helix structure^35^. Our results indicate that the characteristics of the residues forming hydrophobic and hydrophilic regions in the α-helix structure affect solubility of proteins in the *E. coli* expression system.

### Application and definition of the α-helix rule: correcting hydropathy mismatch found in the α-helix by saturated mutagenesis

**Figure 2:**
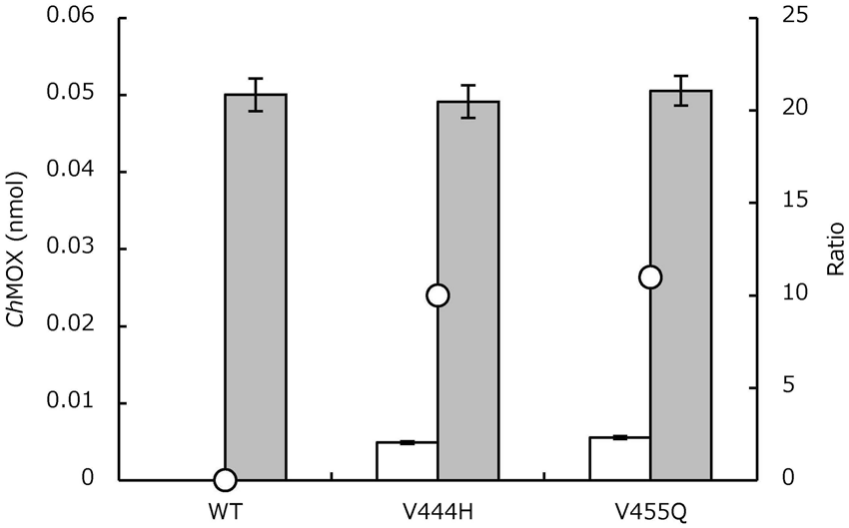
Expression levels of WT and variants of *Ch*MOX. Protein levels were determined by Western blotting. White bar, amounts of soluble enzymes; gray bar, amounts of total enzymes (soluble and insoluble); open circle, ratio (%, soluble/total protein).

Based on the results described in the previous section, we assumed that correcting the hydropathy of the residues in the α-helix structure would enhance protein solubility (α-h﻿elix rule). Therefore, we conducted a more detailed investigation by saturation mutagenesis of *Ch*MOX and *At*ADC, which have different solubility characteristics. In *Ch*MOX, Val444 is located in the hydrophilic region of the α-helix structure (Fig. 1A). Accordingly, we measured the enzyme activity of the Val455 (Fig. 1B) and Val444 (Fig. 1C) mutants (Table S2). For Val455, we obtained nine active mutants, in six of which the Val residue was mutated to a hydrophilic residue (Glu, Gln, Asp, Asn, Lys, or Arg; Fig. 1B). For Val444, similar trends were obtained: thirteen mutations increased *Ch*MOX activity relative to the WT; in nine of these mutants, the Val was substituted with a hydrophilic residue (Fig. 1C). Next, we determined the expression levels of *Ch*MOX and its variants by Western blotting with anti-His-tag antibody (Fig. 2). The total amounts of *Ch*MOX protein (soluble plus insoluble) were similar among the WT and variants, but the soluble amounts of the protein were elevated in the variants, in parallel to their enzyme activities.

In the case of *At*ADC, ADC activity was obtained by mutation of Lys441 to hydrophobic amino acids such as Ala, Leu, or Val (Fig. 1F). Mutation of Leu435 to hydrophilic amino acids (His, Glu, Gln, Asp, or Asn) also showed ADC activity (Fig. 1E). The results indicate that mutation of the residues in the α-helix structure to fit the hydropathy to the surrounding environment is effective for improving protein solubility.

**Figure 3:**
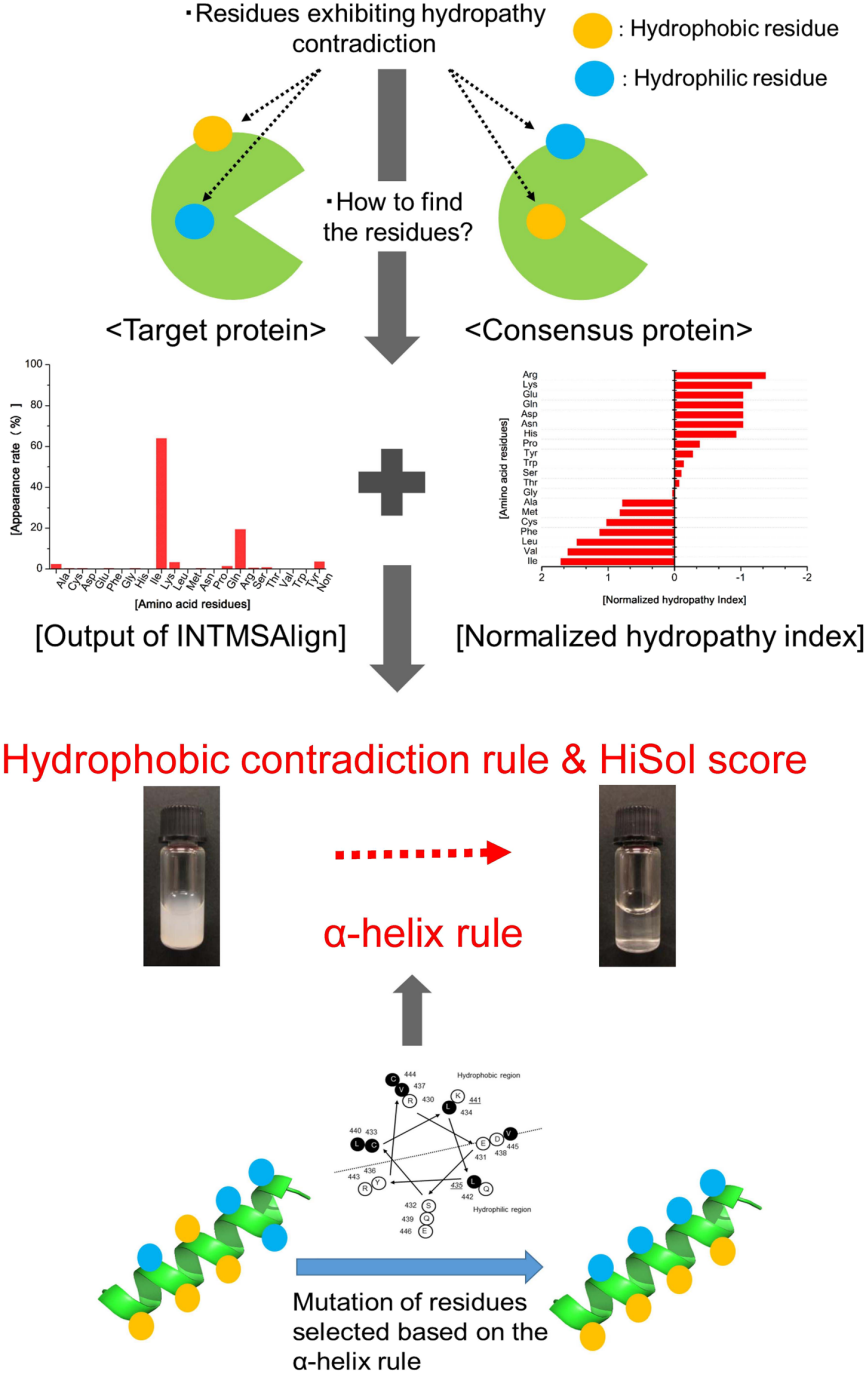
Schematic model for selection of candidate residues for aggregation hotspots. Schematic model based on the hydropathy contradiction rule and the definition of the HiSol score (upper) and the α-helix rule (lower). Hydrophobic and hydrophilic residues are shown as orange and blue filled circles, respectively.

Taken together, these results indicate that soluble and active expression of recombinant proteins in the *E. coli* expression system can be achieved by site-directed mutagenesis correcting the hydrophobicity of residues on α-helix structures. Following this rule, solubility can be enhanced by mutating not only from hydrophobic to hydrophilic residues, but also from hydrophilic to hydrophobic residues, in the α-helix region. We termed this strategy for improving solubility the “α-helix rule” (Fig. 3).

### Rule 2: Identifying aggregation hotspots based on the HiSol score, derived from the successful example of *Me*HNL H103X

In folded proteins, hydrophobic and hydrophilic residues are generally located in the protein core and at the surface, respectively^36^. Thus, residues that fail to obey this rule, such as hydrophilic residues in the interior or hydrophobic residues in the exterior of the protein, often destabilize a protein^37^ or decrease the efficiency of both *in vivo* and *in vitro* folding^38^. Because mutation of residues to conform to these rules is expected to increase folding efficiency and solubility, we identified hotspot residues based on the consensus design method, which improves protein function by mutating certain residues of the target protein to residues that are highly conserved in proteins of the same family. The consensus residues could be identified utilizing INTMSAlign^19^. Next, we calculated HiSol scores for each residue of the target protein utilizing equation (1) in Materials and Methods; here, no 3D structural information (such as PDB data) is required for calculation of the HiSol score. In this calculation, the score would be negative if the residue of the target protein was hydrophilic and the residue of the consensus protein is hydrophobic, and *vice versa* in the case of a positive HiSol score. Thus, the score identifies residues with a contradiction in hydrophobicity by comparing them with their consensus amino acid residues.

We first investigated whether the score could identify aggregation hotspots with reference to the study of *Me*HNL, which was solubilized by site-directed mutagenesis of His103 to hydrophobic residues, such as Val, Leu and Ile, by Asano *et al*.^18^; thus, His103 is one of the hotspots in *Me*HNL. The HiSol score of *Me*HNL was calculated under the runtime parameters suggested in Table S3. The 103^rd^ residue had the most negative score among the 259 total residues of *Me*HNL. Therefore, we concluded that the HiSol score can suggest aggregation hotspots and candidate amino acids for mutation, based solely on the primary sequence (Table 1).

**Table 1:** HiSol scores and conserved residues corresponding to mutated residues in *Me*HNL, *Su*PDH, *Ch*MOX, *At*ADC, and *Mp*LUC.

Enzyme	Target residue	How residues were identified	HiSol score (Ranking)^a^	Conserved residues^b^	Position^c^	Result
*Su*PDH	Lys148	Hydropathy contradiction rule ^d^	−2.748 (1^st^ score)	Ile (64%), Val (21%), Leu (9%)	Coil	Negative
	Gln225		−2.232 (2^nd^ score)	Val (73%)	Helix	Positive
	Lys374		−2.023 (3^rd^ score)	Ile (53%)	Coil	Negative
	Gln337		−1.949 (4^th^ score)	Ile (72%)	Helix	Positive
	Gln243		−1.807 (5^th^ score)	Val (62%)	Coil	Negative
*At*ADC	Leu435	α-helix rule^e^	*1.814* (8^th^ score)	His (31%)	Helix	Positive
	Lys441		*−1.334* (11^th^ score)	Leu (21%), Lys (11%)	Helix	False negative
*Me*HNL	His103	Hydropathy contradiction rule^d^	−2.283 (1^st^ score)	Leu (47%), Val (30%)	Sheet	False negative
*Ch*MOX	Val444	α-helix rule^e,﻿f^	*1.834* (10^th^ score)	Glu (9%)	Helix	False negative
	Val455		*0.621* (106^th^ score)	Leu (42%), Ile (18%)	Helix	False negative
*Dm*ODC	Lys117	α-helix rule^e^	*−1.795*(3^rd^ score)	Leu (30.5%), Ala (22.5%), Tyr (13.3%)	Helix	Positive
*Dm*GDH	Val174	α-helix rule^e^	*2.058* (3^rd^ score)	Asp (24.3%), Glu (15.8%),	Helix	Positive
*Mp*LUC	Ile80	Hydropathy contradiction rule on α-helix^g﻿^	1.919 (2^nd^ score)	Lys (53%), Ile (17%)	Helix	Positive
	Arg87		−1.293 (10^nd^ score)	Arg (41%), Lys (20%), Val (11%), Phe (11%)	Helix	Negative
	Ala177		1.361 (7^th^ score)	Asp (57%), Ala (20%), Glu (13%)	Helix	Positive
*h*GH	Leu114	Hydropathy contradiction rule on α-helix^g﻿^	2.042 (1^st^ score)	Lys (41.4%)	Helix	Positive
	Arg95		−1.473 (2^nd^ score)	Ser (41.1%), Val (16.9%)	Helix	Positive
	Leu46		1.463 (3^rd^ score)	Lys (12.2%), Phe (10.7%)	Helix	Positive
	Leu88		1.369 (4^th^ score)	Leu (33.2%), Glu (19.2%), Asn (16.0%)	Helix	Negative
	Phe55		1.319 (5^th^ score)	His (30.6%), Phe (20.2%), Asn (14.9%)	Helix	Positive
	Leu82		1.231 (6^th^ score)	Leu (23.8%), Arg(19.8%), Val (10.0%)	Helix	Negative
	Val97		1.191 (7^th^ score)	Val (28.3%), Glu (16.2%)	Helix	Positive

^a^Ranking of HiSol score is represented for the predicted hotspots. Here, the score bearing negative value would be ranked after lining the scores with descending order, and *vice versa* for the score bearing positive value.

^b^Ratio of conservation > 10%.

^c^Position in secondary structure was predicted by PSIPRED.

^d^“Hydropathy contradiction rule” means that the hotspots were predicted based on the analysis of the HiSol scores.^e^“α-h﻿elix rule” means that the hotspots were predicted from the α-h﻿elix rule according to the hydropathy index of the ﻿residues. The HiSol scores were not used for the prediction, but are shown in italic as references. Thus, the ranking of the HiSol scores are not always high, but distributed sparse (from 3^rd^ to even 106^th^).^f^These are good examples showing the merit of theα-helix rule: even residues with low appearance rates and lower HiSol scores w﻿ere chosen as hotspots (V444E with 10^th^ HiSol score and only 9% appearance rate, and Val455 with 106^th^ HiSol score in *Ch*MOX). ^g^“Hydropathy contradiction rule on α-helix” means that hotspot residues wer﻿e first selected based on HiSol ﻿scores, and then the residues located on the α-helices were selected.

### Application and definition of hydropathy contradiction rule: design of site-directed mutants based on HiSol score

**Figure 4:**
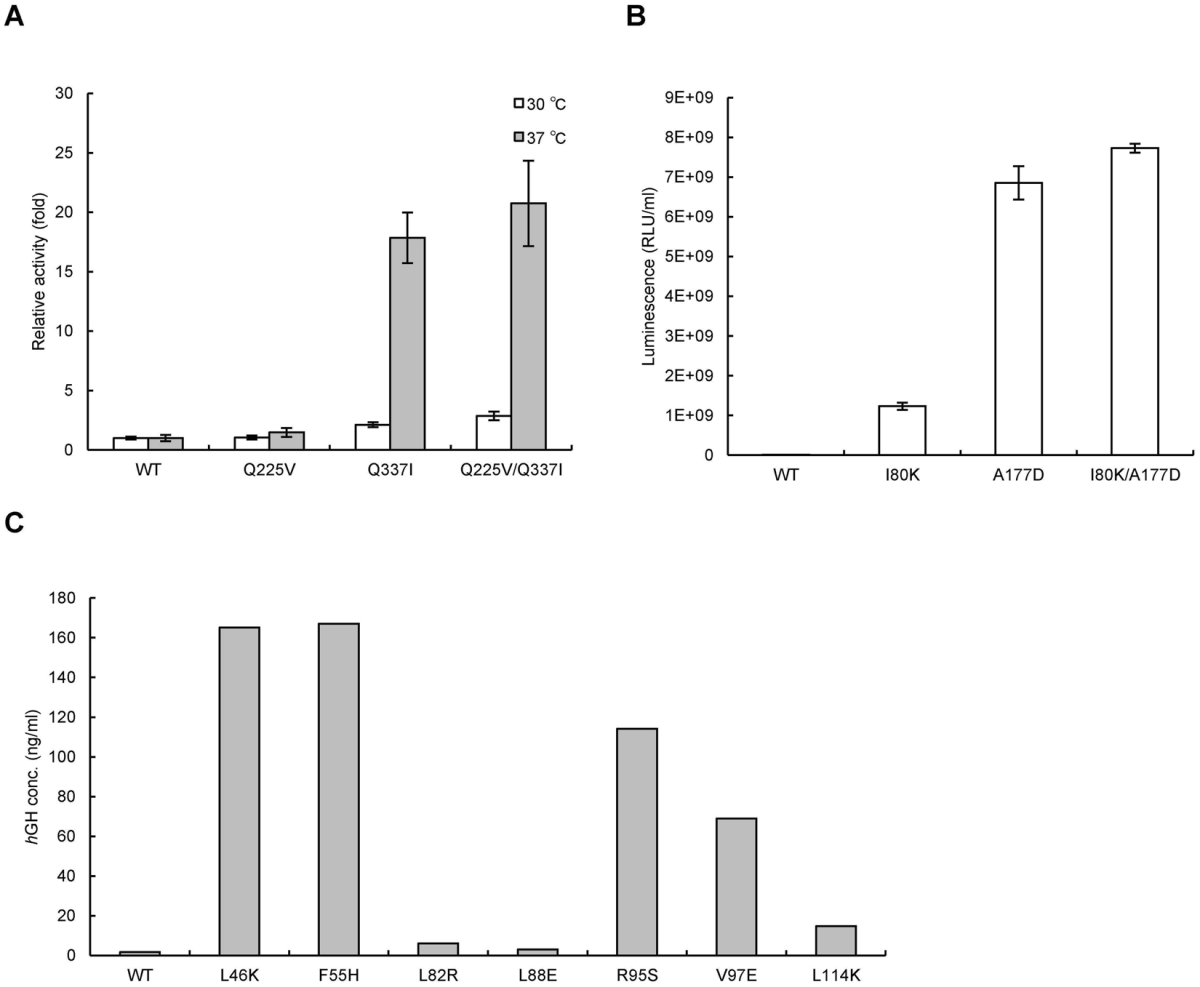
Relative activities of the WT and variants of *Su*PDH (**A**) and *Mp*LUC (**B**). Enzymatic activities of *Su*PDH WT and its variants (**A**). White bar, induction at 30 °C; gray bar, induction at 37 °C. Enzyme activities of *Mp*LUC WT and its variants (**B**). Confirmation of expression levels of WT and variants of *h*GH in *E. coli* (**C**). Protein levels were determined by Western blotting.

Next, to utilize the HiSol score to identify hotspots in various proteins, we applied this method to *Su*PDH. Based on an analysis of the 379 residues of *Su*PDH, we selected the top five residues with negative HiSol score (148, 225, 243, 337, and 374) as candidate hotspots (Table 1). We performed site-directed mutation of these five residues in *Su*PDH (Table S1, P15–19). Three mutants, Q225V, Q337I and Q225V/Q337I, all of which were located in α-helix regions, were obtained as soluble forms with enzyme activity. When these mutants were incubated at 30 °C, the relative activities of Q225V, Q337I, and Q225V/Q337I were approximately 1.1-, 2.1- and 2.9-fold higher than that of WT (white bar, Fig. 4A). By contrast, when the incubation was performed at 37 °C, the relative activities of Q225V, Q337I, and Q225V/Q337I were approximately 1.5-, 18-, and 21-fold higher than that of WT (gray bar, Fig. 4A). Thus, the difference in activity among the WT protein and two of the variants was greater at higher cultivation temperatures. These results confirmed that the HiSol score is applicable to other proteins. In addition, although the propensity of the protein to aggregate is higher at 37 °C than at 30 °C, this propensity could be reduced by mutation. The enzymes obtained by the α-helix rule also had high HiSol scores (Hydropathy contradiction rule) (Table 1).

Summarizing the results described in this section, our findings demonstrate that the HiSol score is also effective for the identification of hotspots for improving protein solubility: residues with very different hydrophobicity from the amino acid in the consensus protein, i.e., residues with hydropathy contradiction based on HiSol score, are candidates for aggregation hotspots, and solubility might be improved by correcting such contradictions through site-directed mutagenesis of these residues. The score appeared to change depending on the sequences in the library; therefore, a threshold of the HiSol score must be individually defined in each protein. In this study, the candidate mutation sites were selected according to the following two conditions: when the HiSol scores of more than 10 residues were > 1.0 (condition A), the ten residues with the highest absolute HiSol scores were selected; otherwise, all residues for which HiSol score was > 1.0 were selected as candidates (condition B). We refer to this method the “hydropathy contradiction rule” (Fig. 3).

### Application of hydropathy contradiction rule on α-helix

In the previous sections, we developed two different methods, the α-helix rule and hydropathy contradiction rule, to increase the solubility of insoluble proteins. Based on these two rules, the following procedure was applied to *Mp*LUC and *h*GH: first, candidate residues were selected based on the hydropathy contradiction rule, and then the residues satisfying the α-helix rule or placed in the α-helix were chosen as the final candidate residues.

In the case of *Mp*LUC, three residues, Ile80, Arg87 and Ala177, were identified as strong candidates; all of these residues were located on the α-helix structure (Fig. S2C and D) and had high HiSol scores (>1.0). Furthermore, the residues were mutated to consensus residues (I80K, R87V, and A177D). Two of the mutants I80K and A177D, enhanced solubility, and the activities of the mutant proteins were more than 10-fold higher than that WT (Table 2, and Fig. 4B). Furthermore, luminescence could be observed in the variants, but not in the WT protein. The WT protein was produced by induction at low temperature. The variants were purified, and their circular dichroism (CD) and luminescence were analyzed after heat treatment at various temperatures. Based on the CD spectrum (Fig. S3C,D) and enzyme activities (Fig. S4), the thermal stability of the two A177D variants was similar to that of the WT protein.

**Table 2:** Comparison of the activities of WT and variants of *Ch*MOX, *At*ADC, and *Mp*LUC.

	*Ch*MOX				*At*ADC			*Mp*LUC		
	WT	Refolded WT	V444H^a^	V455Q^a^	WT	L435H^a^	K441L^a^	WT	I80K^a^	A177D^a^
Total activity (U/mL, or RLU^b^)	ND	0.13	0.61	0.63	ND	0.039	0.043	1.1 × 10^8^	1.2 × 10^9^	2.8 × 10^9^
Soluble protein (mg/mL)	ND	0.01	0.05	0.045	ND	0.021	0.021	0.001	0.01	0.02
Specific activity (U/mg, or RLU/mg^c^)	ND	13	12.2	14	ND	1.9	2.0	1.1 × 10^11^	1.2 × 10^11^	1.4 × 10^11^

^a^Highly soluble.

^b^U/mL or RLU of cell-free extract prepared from a 1-mL LB culture in triplicate; ND, not determined.

^c^Specific activity (U/mg) of these enzymes compared to their purified forms.

In the case of *h*GH, seven residues (L46K, F55H, L82R, L88E, R95S, V97E, and L114K) were identified as candidates, and the expression levels of five mutants (L46K, F55H, R95S, V97E, and L114K) were more than 10-fold higher than that of WT (Fig. 4C).

Thus, using this approach, the solubilities of *Mp*LUC and *h*GH were improved for a total of seven out of ten predicted candidates. These results support the idea that this approach, in which hydrophilic residues in hydrophobic regions in α-helix structures are mutated to hydrophobic amino acids (and *vice versa*) is effective for soluble expression of heterologous proteins in *E. coli*. In addition, the assignment of aggregation hotspots by the combinational approach did not require a large number of homologous sequences of the target protein; in fact, the solubilization of *Mp*LUC was successful using only 37 sequences (Table S3). We expect that this rational approach to mutagenesis will be applicable to heterologous expression of various inactive, aggregated, and rarely studied proteins.

## Discussion

At present, the improvement of protein solubility remains a difficult problem because many factors affect solubility, including the foldability and stability of the proteins, as well as the cultivation temperature. Given this situation, the development of general methods would be of great value to efforts aimed at expanding the applications of enzymes. To address this issue, we derived rules for improving protein solubility based on the previous observation that *Me*HNL could be expressed as an active soluble form by mutation of His103.

In this study, we derived two rules, the α-helix rule and the hydropathy contradiction rule, to identify aggregation hotspots, and then developed new methods for improving protein solubility using these rules. Both methods have the advantage that they require only primary structure data, but not three-dimensional structure information. In addition, the method based on the α-helix rule has another advantage: it can be applied to proteins without similar sequences, but only if they have α-helix structures. On the other hand, the method based on the hydropathy contradiction rule can be applied to proteins without α-helix structure. In addition, this method can be applied to identify both mutation sites and candidate replacement amino acids without utilizing secondary structure information (as in the α-helix rule); however, this method requires similar sequences obtained by Blastp search of databases. Proper application of these methods to target proteins increases the probabilities of obtaining solubilized proteins.

We also further considered how insoluble proteins could be solubilized by correcting hydropathy contradiction. Ventura reported that there is a positive correlation between protein stability and solubility^2^. However, we observed no significant correlation among the mutants obtained in this study. The thermal stability of the mutants barely increased, although solubility was enhanced by mutation of hydrophobic residues to hydrophilic residues: the T_m_ values of *Ch*MOx were 67 °C (WT) and 67.3 °C (V455E) (Fig. S3B), and those of *Mp*LUC were 86.8 °C (WT) and 87.5 °C (A177D) (Fig. S3D). Likewise, mutation of hydrophilic residues to hydrophobic residues barely improved the stability: the T_m_ values of *Dm*ODC were 67.8 °C (WT) and 67.0 °C (K117L) (Fig. S3E), and those of *At*ADC were 60.0 °C (WT) and 59.8 °C (K441L) (Fig. S3F). For *Me*HNL, the T_m_ value of H103L, a highly soluble mutant, was about 5 °C lower than that of WT^18^. These results suggested that mutations that enhance protein solubility do not always improve protein stability. One hypothesis that would explain the enhancement of solubility by correction of the hydrophobic contradiction is that the mutations improve the efficiency of protein refolding. However, we acknowledge that the data are not sufficient to prove this hypothesis; experiments to test this idea are currently underway.

**Figure 5:**
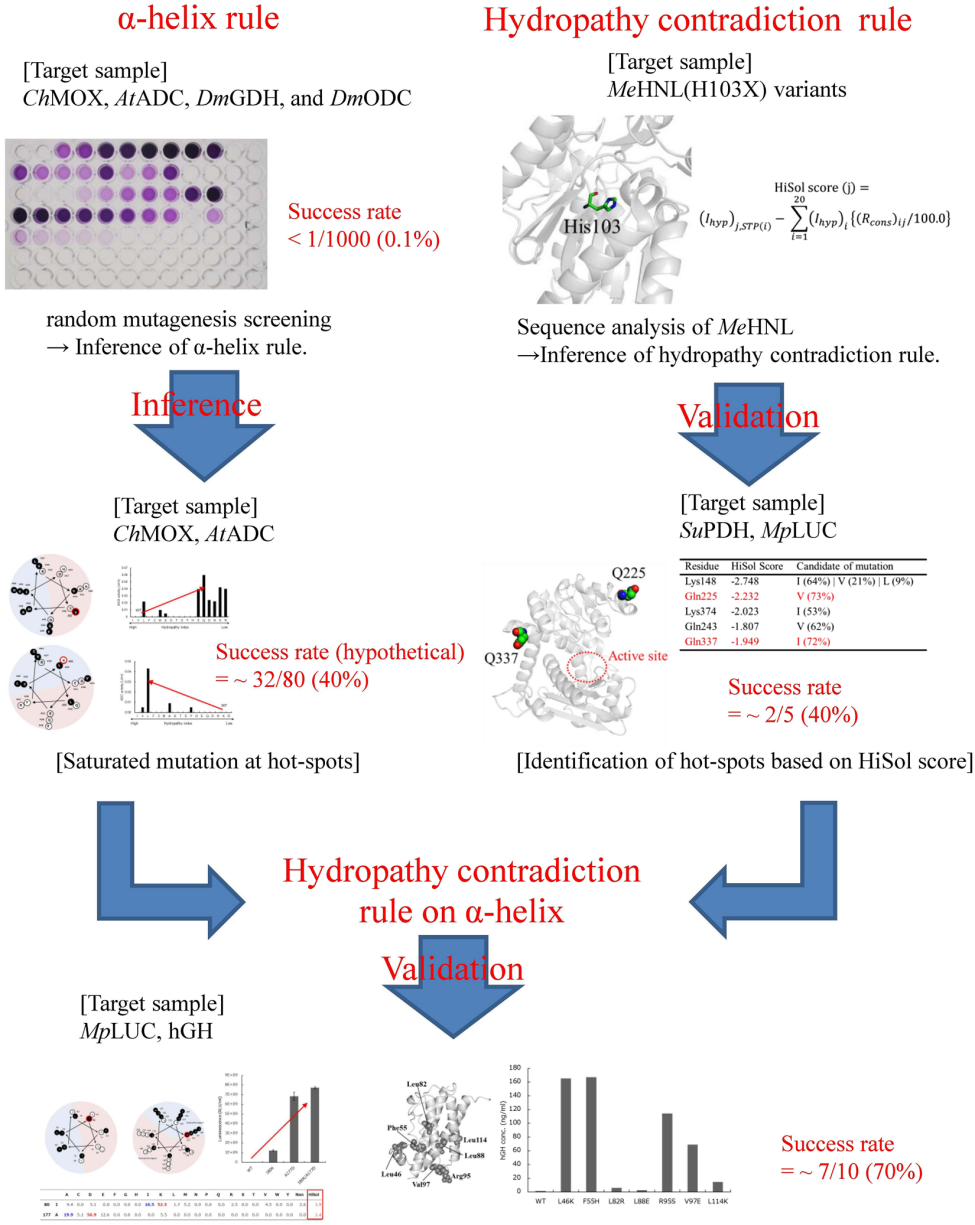
Schematic of inference and validation of the α-helix rule and hydropathy contradiction rule. For the α-helix rule, we made inferences based on positive variants of four enzymes obtained by random mutagenesis screening. The rule was inferred by saturating mutagenesis at these sites in *Ch*MOX (Val455) and *At*ADC (Leu435). On the other hand, for the hydropathy contradiction rule, we made inferences based on sequence analysis of *Me*HNL. The rule was validated by identifying five candidate hotspots in *Su*PDH. Finally, we evaluated the effectiveness of the rules by applying the method to *Mp*LUC and *h*GH.

A flowchart of the inference and validation of the two rules and the success rate of obtaining soluble variants is summarized in Fig. 5. By random mutagenesis screening, the success rate was less than 0.1% for five enzymes: *Ch*MOX, *At*ADC, *Dm*GDH, *Dm*ODC, and *Su*PDH (Fig. 5). In particular, in the screen for *Su*PDH, no positive variant could be identified, despite the fact that more than 1000 colonies were analyzed. On the other hand, the success rate was improved to ~40% (i.e., the false positive rate was ~60%) using either the α-helix rule or hydropathy contradiction rule (Fig. 5). Using the α-helix rule, we identified four candidate residues and obtained a total of 32 solubilized variants (Fig. 1B,C,E,F), out of a total of 80 possible variants, by saturation mutagenesis at those residues; thus, the success rate (hypothetical) was about 40% (Fig. 5). Here, the rate is a hypothetical value because the candidate residues were not assigned *ab initio* utilizing the α-helix rule. By applying the hydropathy contradiction rule, we identified five candidate variants of *Su*PDH (K148I, Q225V, K374I, Q243V and Q337I), and obtained two solubilized variants (Q225V and Q337I); thus, the success rate was 40% (Fig. 5). Furthermore, selection of candidate residues on an α-helix that satisfy the hydropathy contradiction rule improved the success rate to ~70% (Fig. 5) (i.e., a false-positive rate of ~30%); a total of 10 variants were predicted as candidates for *h*GH and *Mp*LUC, and seven variants exhibited improved solubility (Table 2 and Fig. 4B).

Next, we considered the false-negative rate of the approach. Here, we regarded the soluble variants indicated in Table 1 that satisfied only one of the two rules as “false-negative”. In this situation, the false-negative rate was ~27%: four of fifteen variants that exhibited improved solubility (Table 1) satisfied either the α-helix rule or hydropathy contradiction rule. This implies that some insoluble proteins may be solubilized by mutating specific residues to satisfy either, but not necessarily both, of the two rules.

Taken together, the findings of this study demonstrate that we have identified an effective method for identifying aggregation hotspots. Of course, certain challenges remain; for example, the existence of correlated residues should also be considered in the course of identification in order to reduce the false-positive rates of the methods. Furthermore, because the efficiency of protein folding is decreased by mutations that interfere with intermolecular protein–protein interactions, and improvement was achieved only after simultaneous mutation of two residues, as in the case improved protein stability^39^, mutation of one of the correlation residues may not be sufficient to improve protein solubility. Many studies also require application of this method to membrane proteins. Despite these challenges, our methods should have an impact on protein engineering because they enable the generation of solubilized proteins with a high success rate.

## Electronic supplementary material

Supplemental information

## References

[1] Chen R (2012). Bacterial expression systems for recombinant protein production: E. coli and beyond. Biotechnol. Adv..

[2] Ventura S (2005). Sequence determinants of protein aggregation: tools to increase protein solubility. Microb. Cell Fact..

[3] Ventura S, Villaverde A (2006). Protein quality in bacterial inclusion bodies. Trends Biotechnol..

[4] Luan CH (2004). High-throughput expression of C. elegans proteins. Genome Res..

[5] Sorensen HP, Mortensen KK (2005). Soluble expression of recombinant proteins in the cytoplasm of Escherichia coli. Microb. Cell Fact..

[6] Semba H, Ichige E, Imanaka T, Atomi H, Aoyagi H (2008). Efficient production of active form of recombinant cassava hydroxynitrile lyase using Escherichia coli in low-temperature culture. Appl. Microbiol. Biotechnol..

[7] Yang Q, Xu J, Li M, Lei X, An L (2003). High-level expression of a soluble snake venom enzyme, gloshedobin, in E. coli in the presence of metal ions. Biotechnol. Lett..

[8] Garcia-Fruitos E (2007). Divergent genetic control of protein solubility and conformational quality in Escherichia coli. J. Mol. Biol..

[9] Tokuriki N, Tawfik DS (2009). Chaperonin overexpression promotes genetic variation and enzyme evolution. Nature.

[10] Idicula-Thomas S, Balaji PV (2005). Understanding the relationship between the primary structure of proteins and its propensity to be soluble on overexpression in Escherichia coli. Protein Sci..

[11] Magnan CN, Randall A, Baldi P (2009). SOLpro: accurate sequence-based prediction of protein solubility. Bioinformatics.

[12] Smialowski P, Doose G, Torkler P, Kaufmann S, Frishman D (2012). PROSO II–a new method for protein solubility prediction. FEBS J..

[13] Goldenzweig A (2016). Automated structure- and sequence-based design of proteins for high bacterial expression and stability. Mol. Cell.

[14] Campeotto I (2017). One-step design of a stable variant of the malaria invasion protein RH5 for use as a vaccine immunogen. Proc. Natl. Acad. Sci. U S A.

[15] Conchillo-Sole O (2007). AGGRESCAN: a server for the prediction and evaluation of “hot spots” of aggregation in polypeptides. BMC Bioinformatics.

[16] Van Durme J (2016). Solubis: a webserver to reduce protein aggregation through mutation. Protein Eng. Des. Sel..

[17] Sormanni P, Aprile FA, Vendruscolo M (2015). The CamSol method of rational design of protein mutants with enhanced solubility. J. Mol. Biol..

[18] Asano Y, Dadashipour M, Yamazaki M, Doi N, Komeda H (2011). Functional expression of a plant hydroxynitrile lyase in Escherichia coli by directed evolution: creation and characterization of highly in vivo soluble mutants. Protein Eng. Des. Sel..

[19] Nakano, S. & Asano, Y. Protein evolution analysis of S-hydroxynitrile lyase by complete sequence design utilizing the INTMSAlign software. *Sci. Rep*. **5**, doi:10.1038/srep08193 (2015). 10.1038/srep08193 PMC4648443 25645341

[20] Tachibana S, Kuwamori Y, Asano Y (2009). Discrimination of aliphatic substrates by a single amino acid substitution in Bacillus badius and Bacillus sphaericus phenylalanine dehydrogenases. Biosci. Biotechnol. Biochem..

[21] Ishida Y (2016). A sacrificial millipede altruistically protects its swarm using a drone blood enzyme, mandelonitrile oxidase. Sci. Rep..

[22] Laemmli UK (1970). Cleavage of structural proteins during the assembly of the head of bacteriophage T4. Nature.

[23] Sugawara A (2014). Characterization of a pyridoxal-5’-phosphate-dependent L-lysine decarboxylase/oxidase from Burkholderia sp. AIU 395. J. Biosci. Bioeng..

[24] Asano Y, Nakazawa A, Endo K (1987). Novel phenylalanine dehydrogenases from Sporosarcina ureae and Bacillus sphaericus. Purification and characterization. J. Biol. Chem..

[25] Takenaka Y (2008). Two forms of secreted and thermostable luciferases from the marine copepod crustacean, Metridia pacifica. Gene.

[26] Bradford MM (1976). A rapid and sensitive method for the quantitation of microgram quantities of protein utilizing the principle of protein-dye binding. Anal. Biochem..

[27] Matsui D, Asano Y (2015). Heterologous production of L-lysine ε-oxidase by directed evolution using a fusion reporter method. Biosci. Biotechnol. Biochem..

[28] Jones DT (1999). Protein secondary structure prediction based on position-specific scoring matrices. J. Mol. Biol..

[29] Guex N, Peitsch MC, Schwede T (2009). Automated comparative protein structure modeling with SWISSMODEL and Swiss-PdbViewer: a historical perspective. Electrophoresis.

[30] Rice P, Longden I, Bleasby A (2000). EMBOSS: the european molecular biology open software suite. Trends Genet..

[31] Wang H (2010). High-level expression and purification of soluble recombinant FGF21 protein by SUMO fusion in Escherichia coli. BMC Biotechnol..

[32] Kyte J, Doolittle RF (1982). A simple method for displaying the hydropathic character of a protein. J. Mol. Biol..

[33] Nakamura Y, Gojobori T, Ikemura T (2000). Codon usage tabulated from international DNA sequence databases: status for the year 2000. Nucleic. Acids Res..

[34] Tsai CJ, Lin SL, Wolfson HJ, Nussinov R (1997). Studies of protein-protein interfaces: a statistical analysis of the hydrophobic effect. Protein Sci..

[35] Kamtekar S, Schiffer JM, Xiong H, Babik JM, Hecht MH (1993). Protein design by binary patterning of polar and nonpolar amino acids. Science.

[36] Rose G, Geselowitz A, Lesser G, Lee R, Zehfus M (1985). Hydrophobicity of amino acid residues in globular proteins. Science.

[37] Hendsch ZS, Tidor B (1994). Do salt bridges stabilize proteins? A continuum electrostatic analysis. Protein Sci..

[38] Nieba L, Honegger A, Krebber C, Pluckthun A (1997). Disrupting the hydrophobic patches at the antibody variable/constant domain interface: improved in vivo folding and physical characterization of an engineered scFv fragment. Protein Eng..

[39] Sullivan BJ (2012). Stabilizing proteins from sequence statistics: the interplay of conservation and correlation in triosephosphate isomerase stability. J. Mol. Biol..

