# Supplementary material for: Rational identification of aggregation hotspots based on secondary structure and amino acid hydrophobicity
Source: Sci Rep. 2017 Aug 25;7:9558. doi: 10.1038/s41598-017-09749-2 (PMC5573320; doi:10.1038/s41598-017-09749-2)
Supplement: Supplementary file 1 — Supplemental information [file 41598_2017_9749_MOESM1_ESM.pdf]

Rational identification of aggregation hotspots based on secondary structure and amino acid hydrophobicity

Daisuke Matsui[1,2$], Shogo Nakano[1,2$†], Mohammad Dadashipour[1,2], and Yasuhisa Asano[1,2,*]

[1] Biotechnology Research Center and Department of Biotechnology, Toyama Prefectural University, 5180 Kurokawa, Imizu, Toyama 939-0398, Japan.

[2] Asano Active Enzyme Molecule Project, ERATO, JST, 5180 Kurokawa, Imizu, Toyama 939-0398, Japan

[†] Current address: Graduate School of Pharmaceutical and Nutritional Sciences, University of Shizuoka, 52-1 Yada, Suruga-ku, Shizuoka 422-8526, Japan.

Supplemental Information

Screening of soluble variants of 3-hydroxybutyrate dehydrogenase and tryptophan synthase from *Thermus thermophilus* HB8

The expression plasmids of 3-hydroxybutyrate dehydrogenase (*Tt*HBD; GenBank accession number BAD70516.1) and tryptophan synthase (*Tt*TST; GenBank accession number YP_144360.1) from *T. thermophilus* were from RIKEN BioResource Center (Ibaraki, Japan)[1]. These plasmids were transformed into *E. coli* BL21 (DE3), the transformants were grown in LB medium, and the protein expression was induced under the same conditions as described in Materials and Methods. *E. coli* XL-1 Red was used for the random mutagenesis according to the method described in a previous report [2]. The soluble expressions were obtained from each of the two enzymes in SDS-PAGE, and the following amino acid substitutions were identified: A25E in *Tt*HBD, and V136A in *Tt*TST. The mutated residues were located in α-helix structures.

Expression and characterization of *Ch*MOX, *At*ADC, *Dm*GDH, *Dm*ODC, *Su*PDH, and *Mp*LUC

The genes *chmox*, *supdh*, and *mpluc* were obtained from our laboratory stocks, and *atadc*, *dmgdh*, and *dmodc* for the construction of expression plasmids were cloned from the *A. thaliana* or *D. melanogaster* cDNA library. The sequence sizes of *Ch*MOX, *At*ADC, *Dm*GDH, *Dm*ODC, *Su*PDH and *Mp*LUC are 584, 702, 535, 394, 379, and 208 residues, respectively, and the theoretical molecular weights are 63,800, 76,200, 59,900, 44,200, 41,300, and 22,500, respectively (Fig. S1 and S2). These genes were expressed using the pET, pUC or pCold system in *E. coli* BL21 (DE3) and were cultivated and induced with IPTG. Most of these genes were expressed in the insoluble fractions in SDS-PAGE assays. No activity of *Ch*MOX, *At*ADC, *Dm*GDH, *Dm*ODC or *Mp*LUC could be detected in the crude extracts (soluble fractions), and

the activities of *Su*PDH were very low.

*Ch*MOX and *Mp*LUC have no rare codons, but there are six rare codons (Arg200, Arg229, Arg268, Arg358, Arg512, and Arg630) in *At*ADC, three rare codons (Arg18, Arg472, and Arg513) in *Dm*GDH, three rare codons (Arg54, Arg71, and Arg288) in *Dm*ODC, and one rare codon (Arg233) in *Su*PDH. Because the enzyme was produced in the *E. coli* BL21 (DE3) expression system, the gene sequences do not affect the production of the mRNAs for the enzymes. The same results were obtained in the *E. coli* BL21-CodonPlus (DE3)-RIL strain (Stratagene, CA, USA), which contains extra copies of the *E. coli argU*, *ileY*, and *leuW* tRNA genes. It is suggested that expression speed, translational factors, chaperone recognitions, or posttranslational modifications such as glycosylation affect the soluble and active expression of the genes.

CD spectra of *Ch*MOX WT and its variants

The CD spectra of the *Ch*MOX, *Mp*LUC, *Dm*ODC, and *At*ADC WT and its variant V455D were measured utilizing a Jasco J-715CD spectrometer (Fig. S3). The buffer contained 10 mM potassium phosphate (pH 7.0) and 50 mM sodium chloride, and 0.1 mg/ml of the enzyme was utilized in the measurement. Far-ultraviolet spectra were recorded from 195 to 280 nm according to the method described in a previous report[3].

Soluble expression of carbonyl reductase from yeast *Ogataea polymorpha* NBRC 0799

For the expression of the carbonyl reductase (*Og*CRD; GenBank accession number LC176491) gene (*ogcrd*), the already constructed plasmid pET-11a-*ogcrd*, was used in this study. The plasmids were transformed into *E. coli* BL21 (DE3). The transformants were grown in LB medium and the protein expression was induced under the same conditions as described

in Materials and Methods. The enzyme activity for the reduction of acetone was assayed at 30ºC by measuring the oxidation of NADH to NAD^+ at pH 6.0. One unit of enzyme activity was defined as the amount of enzyme catalyzing the oxidation of one micromole of NADH per min.

Soluble expression of human crystalline aldehyde dehydrogenase and growth hormone

A cDNA of human crystalline aldehyde dehydrogenase (ALDH3A1; GenBank accession number NP_000682.3) was synthesized and amplified using Tks Gflex DNA polymerase and the primers P26 and P27 listed in Table S1. After digestion of pET-15a by *Nde*I and *Bam*HI, the amplified ALDH3A1 gene was ligated to pET-15a using an In-Fusion HD Cloning Kit. A cDNA of human growth hormone (GenBank accession number KJ608193, hGH) was synthesized and amplified using Tks Gflex DNA polymerase and the primers P26 and P27 listed in Table S1. After digestion by *Nde*I, the amplified gene was ligated to pET15b with an In-Fusion HD Cloning Kit. The plasmids were transformed into *E. coli* BL21 (DE3). The transformants were grown in LB medium and the protein expression was induced under the same conditions as described in Materials and Methods. LDH3A1 activity was measured by monitoring the production of β-NADH at 340 nm, following the procedure described in previous reports [4]. The soluble expression levels of hGHs were determined by hGH ELISA kit (Roche, Mannheim, Germany).

From the analysis of hGH, seven residues, Leu46, Phe55, Leu82, Leu88, Arg95, Val97, and Leu114, which were on α-helices and had high or low HiSol scores (more than 1.0 or less than -1.0), were selected as aggregation hot-spots (Table 1). As expected, the mutations L46K, F55H, L82R, L88E, R95S, V97E, and L114K enhanced the solubility compared with WT (Fig. 4C).

Additional information

We declare that there are no competing financial interests in this work.

References

1 Yokoyama, S. *et al.* Structural genomics projects in Japan. *Nat Struct Biol* **7 Suppl**, 943-945 (2000).

2 Matsui, D. & Asano, Y. Heterologous production of L-lysine ε-oxidase by directed evolution using a fusion reporter method. *Biosci Biotechnol Biochem* **79**, 1473-1480 (2015).

3 Nakano, S. & Asano, Y. Protein evolution analysis of *S*-hydroxynitrile lyase by complete sequence design utilizing the INTMSAlign software. *Sci. Rep.* **5** (2015).

4 Voulgaridou, G.-P., Mantso, T., Chlichlia, K., Panayiotidis, M. I. & Pappa, A. Efficient *E. coli* expression strategies for production of soluble human crystallin ALDH3A1. *PloS one* **8**, e56582 (2013).

Supplemental Figures

Figure S1. Amino acid sequences of *Ch*MOX, *At*ADC, *Dm*GDH, *Dm*ODC, *Su*PDH, and *Mp*LUC (A, C, E, G, I, K, respectively) and their homology model structures generated using SWISS-MODEL (B, D, F, H, J, respectively, omitting *Mp*LUC).

The residues in the sequences are highlighted based on the following factors: the α-helix structure in the sequence (underlined), the target α-helix structures (bold font), and the mutated residues (squared). The structure of *Mp*LUC is a novel one that could not be predicted.

Figure S2. Depiction of helical wheels of target α-helices of *Su*PDH and *Mp*LUC. Helical wheels of target α-helices: residues 218-227 (EQAIADIQKL) of *Su*PDH (A), residues 317-340 (PARVLAKTENIYTSLLEVFHQAEQ) of *Su*PDH (B), residues 76-89 (LEVLIEMEANARKA) of *Mp*LUC (C), and residues 176-201 (SALLKKWLPDRCASFADKIQSEVDNI) of *Mp*LUC (D)). The hydrophobic amino acids, the hydrophilic amino acids, and the target amino acids are represented by black filled circles, white circles and underlined residue numbers, respectively.

Figure S3. Comparisons of the CD spectra of WT proteins (filled circle) and variants (open circles).

CD spectra of the refolded WT of *Ch*MOX and the V455E variant are shown in A, and the changes in CD at 222 nm measured after heat treatment are shown in B. The CD spectra of the refolded WT of *Mp*LUC and the A177D variant and the changes in CD at 222 nm induced by heat treatment were also measured (C, D). The changes in CD at 222 nm induced by heat treatment were measured in *Dm*ODC WT and K117L (E) and *At*ADC WT and K441L (F).

Figure S4. Comparisons of the thermal stability of *Mp*LUC WT proteins (filled circle), the I80K, and the A177D variant (filled triangle).

The each luminescence was measured after heat treatment for 30 min.

Supplemental Tables

Table S1. Designed oligonucleotides used to perform random mutagenesis and site-directed mutagenesis.

Table S2. Enzyme activity measurement of the Val444 and Val455 variants of *Ch*MOX and the Leu435 and Lys441 variants of *At*ADC generated by saturation mutagenesis.

Table S3. Run time parameters of INTMSAlign for *Ch*MOX, *At*ADC, *Dm*GDH, *Dm*ODC, *Su*PDH and *Mp*LUC.

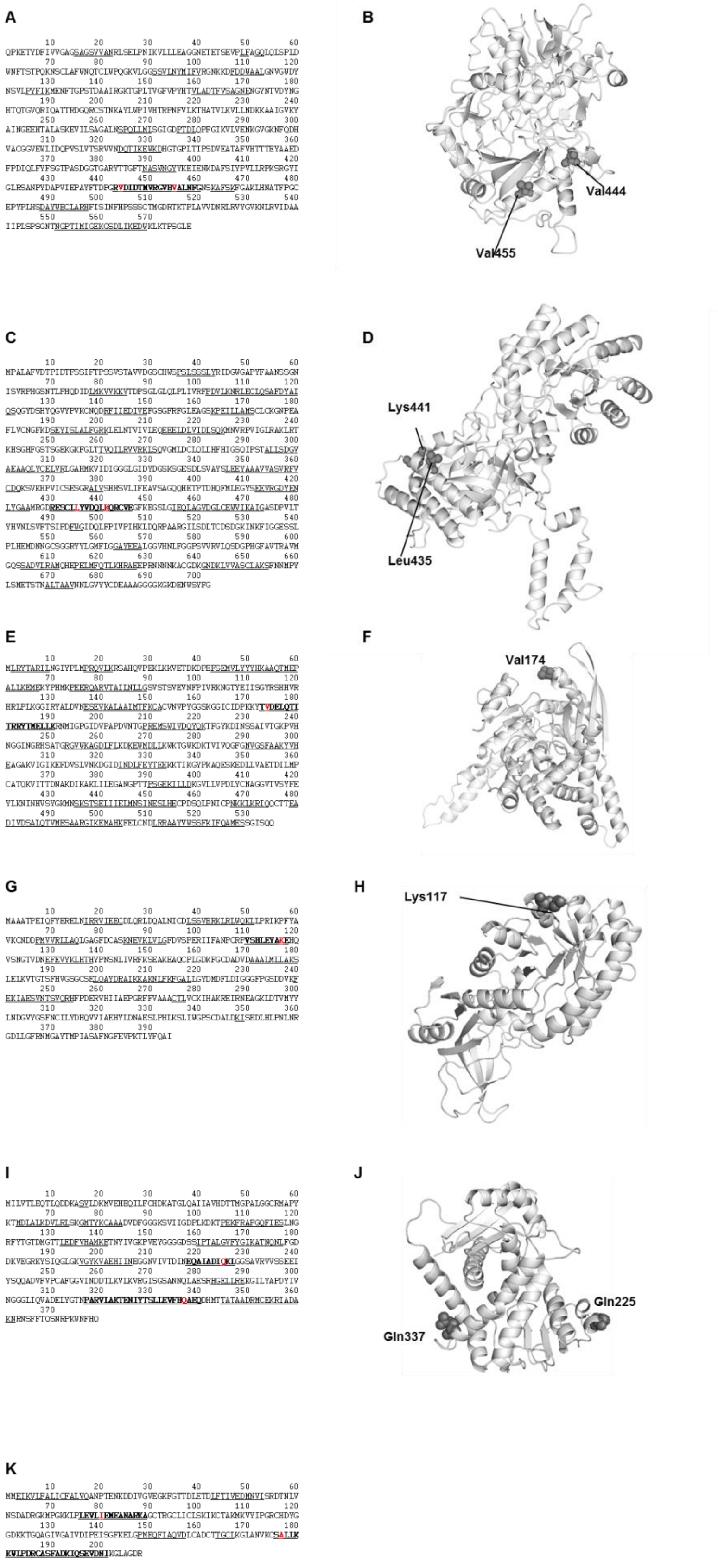
A
B
Val444
Val455
C
D
Lys441
Leu435
E
F
Val174
G
H
Lys117
I
J
Gln225
Gln337
K

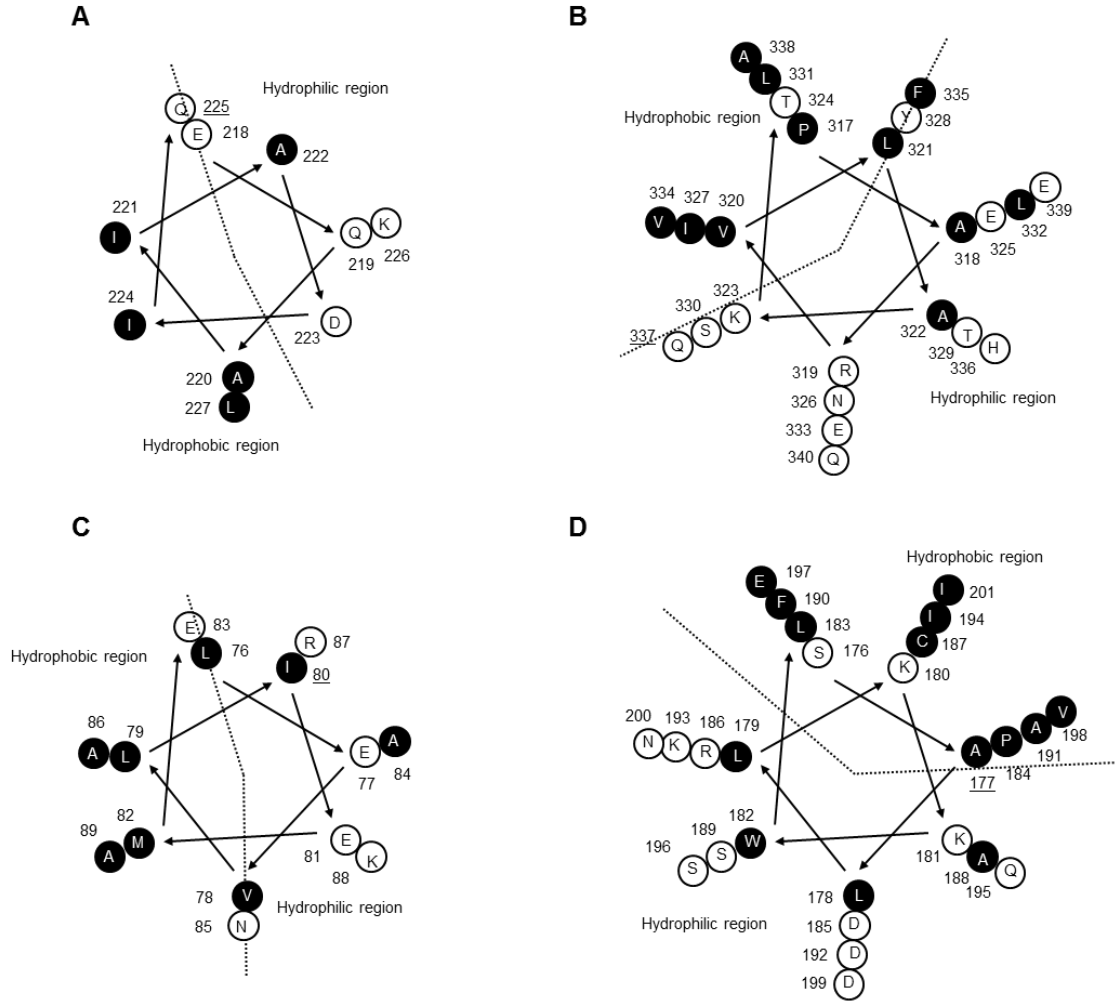
A
Hydrophilic region
Q 225
E 218
A 222
I 221
Q 219
K 226
I 224
D 223
A 220
L 227
Hydrophobic region
B
A 338
L 331
T 324
P 317
Hydrophobic region
F 335
Y 328
L 321
V 334
I 327
V 320
A 318
E 325
L 332
E 339
K 323
S 330
Q 337
A 322
T 329
H 336
R 319
N 326
E 333
Q 340
Hydrophilic region
C
E 83
L 76
R 87
I 80
Hydrophobic region
A 86
L 79
E 77
A 84
A 89
M 82
E 81
K 88
V 78
N 85
Hydrophilic region
D
Hydrophobic region
E 197
F 190
L 183
S 176
I 201
I 194
C 187
K 180
N 200
K 193
R 186
L 179
A 177
P 184
A 191
V 198
W 182
S 189
S 196
K 181
A 188
Q 195
L 178
D 185
D 192
D 199
Hydrophilic region

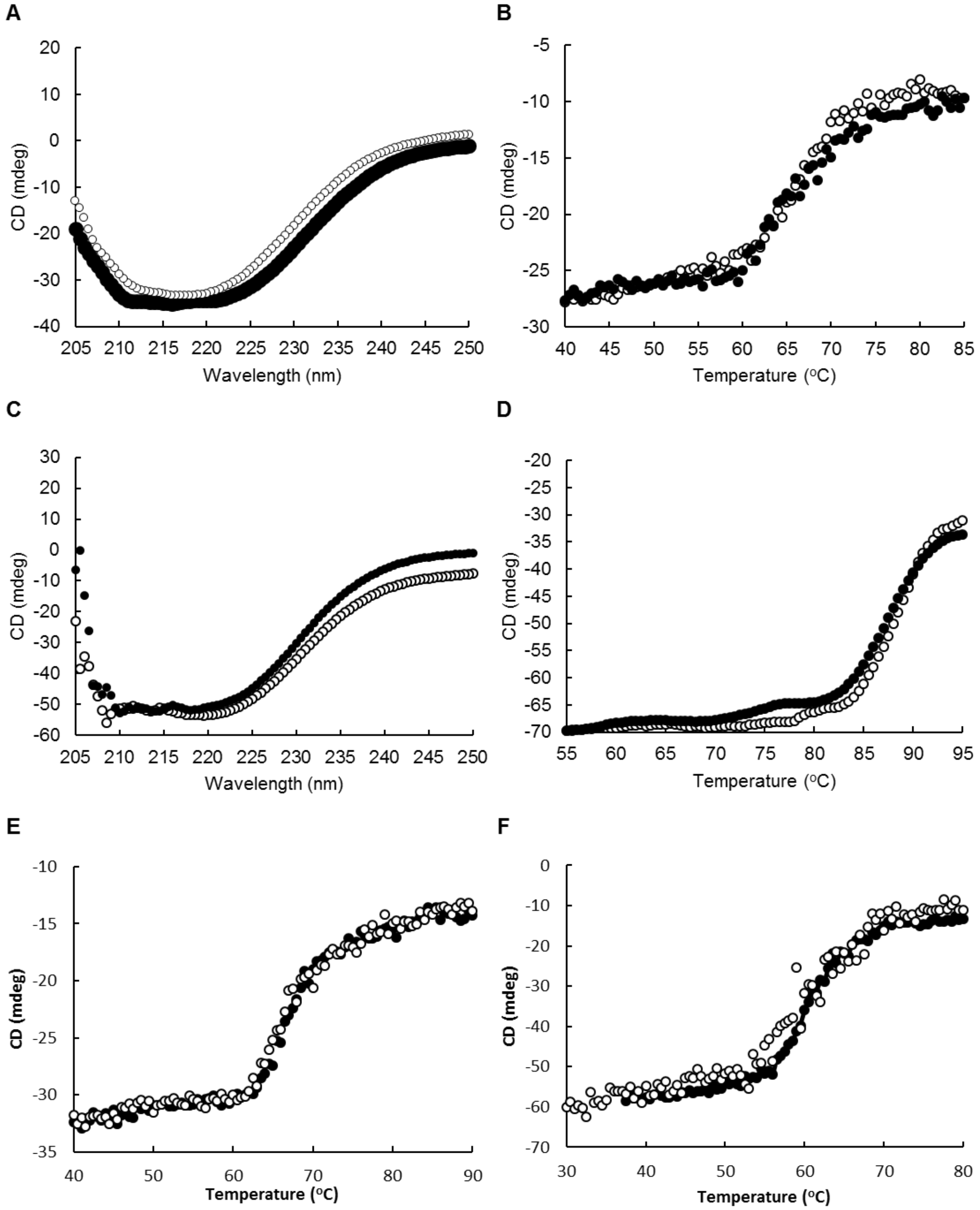
A
B
C
D
E
F
CD (mdeg)
Wavelength (nm)
Temperature (°C)

Fig. S4

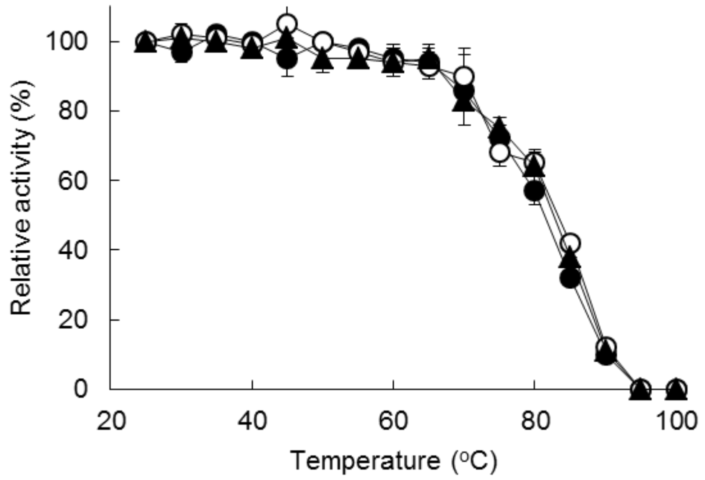
Relative activity (%)
0
20
40
60
80
100
20
40
60
80
100
Temperature (oC)

Table S1. Oligonucleotides used in this study.

Primer	Comments[a]
P1	5’-GCGCATATGAATCCGACCGAAAACAAAGATG-3’, Amplification of *Mp*LUC gene
P2	5’-CGCCTCGAGTTAACGATCGCCTGCCAGGCCTTT-3’, Amplification of *Mp*LUC gene
P3	5'-GGAGATATACATATGCCTGCTCTAGCTTTTGTTGA-3', Amplification of *At*ADC gene
P4	5'-TTAGCAGCCGGATCCTCAACCGAAATAAGACCAAT-3', Amplification of *At*ADC gene
P5	5'-AGAAGGAGATATACATATGCTTCGTTATACGGCACGGATT-3', Amplification of *Dm*GDH gene
P6	5'-TTTGTTAGCAGCCGGATCCCTACTGTTGGGAAATTCCGGAGC-3', Amplification of *Dm*GDH gene
P7	5'-AGAAGGAGATATACATATGGCGGCCGCTACCCCTGAAAT-3', Amplification of *Dm*ODC gene
P8	5'-TTTGTTAGCAGCCGGATCCTCATATAGCTTGGAAGTACAGGG-3', Amplification of *Dm*ODC gene
P9	5'-ACCGACCCTGGAAGANNSGACATTGATACGAT-3', Saturation site-directed mutagenesis at Val444 of *Ch*MOX
P10	5'-ATCGTATCAATGTCSNNTCTTCCAGGGTCGGT-3', Saturation site-directed mutagenesis at Val444 of *Ch*MOX
P11	5'-GTTCGAGGCGTACACNNSGCTCTTAACTTTGGA-3', Saturation site-directed mutagenesis at Val455 of *Ch*MOX
P12	5'-TCCAAAGTTAAGAGCSNNGTGTACGCCTCGAAC-3', Saturation site-directed mutagenesis at Val455 of *Ch*MOX
P13	5'-CGTGAAAGCTGCTTGNNSTATGTTGATCAGCTG-3', Saturation site-directed mutagenesis at Leu435 of *At*ADC
P14	5'-CAGCTGATCAACATASNNCAAGCAGCTTTCACG-3', Saturation site-directed mutagenesis at Leu435 of *At*ADC
P15	5'-ATGTTGATCAGCTGNNSCAGAGATGTGTTGAAG-3', Saturation site-directed mutagenesis at Lys441 of *At*ADC
P16	5'-CTTCAACACATCTCTGSNNCAGCTGATCAACAT-3', Saturation site-directed mutagenesis at Lys441 of *At*ADC
P17	5’-GTGGGCVTCCCGGTCGAATATGGTGGCGGT-3’, Amino acid substitution at Lys148 to Ile, Val or Leu of *Su*PDH
P18	5’-GATATTGTGAAGCTCGGTGGAAGCGCTGTC-3’, Amino acid substitution at Gln225 to Val of *Su*PDH
P19	5’-AGTCAGGYAGCAGATGTTTTTGTTCCTTGT-3’, Amino acid substitution at Gln243 to Val or Ala of *Su*PDH
P20	5’-TTCCATATCGCAGAACAGGATCATATGACA-3’, Amino acid substitution at Gln337 to Ile of *Su*PDH
P21	5’-AACCGACCGRTATGGAATTTTCATCAGTAA-3’, Amino acid substitution at Lys374 to Ile or Val of *Su*PDH

P22	5'-CCGCTGGAAGTTCTGAAAGAAATGGAAGCAAAT-3', Amino acid substitution at Ile80 to Lys of *Mp*LUC
P23	5'-ATTTGCTTCCATTTCTTTCAGAACTTCCAGCGG-3', Amino acid substitution at Ile80 to Lys of *Mp*LUC
P24	5'-AATGTTAAATGTAGCGATCTGCTGAAAAAATGG-3', Amino acid substitution at Ala177 to Asp of *Mp*LUC
P25	5'-CCATTTTTTCAGCAGATCGCTACATTTAACATT-3', Amino acid substitution at Ala177 to Asp of *Mp*LUC
P26	5’-GCTAATTTTGCTCATATGGCTGCCGCGCGGCACCA-3’, Amplification of ALDH3A gene
P27	5’-ATGACCCAGCATTAAGGATCCGGCTGCTAACAAAG-3’, Amplification of ALDH3A gene
P28	5’-CGCGGCAGCCATATGTTTCCGACCATTCCGCTGAGCC-3’, Amplification of hGH gene
P29	5’-TTAGCAGCCGGATCCTTAAAAACCACAGCTACCTTCAAC-3’, Amplification of hGH gene

[a] Mutation sites are underlined.

Table S2. Saturation mutagenesis at Val444 and at Val455 of *Ch*MOX and at Leu435 and at Lys441 of *At*ADC

	*Ch*MOX Val444			*Ch*MOX Val455			*At*ADC Leu435			*At*ADC Lys441		
	Total activity (U/ml)	Soluble protein (mg/ml)[a]	Total specific activity (U/mg)	Total activity (U/ml)	Soluble protein (mg/ml)	Total specific activity (U/mg)	Total activity (U/ml)	Soluble protein (mg/ml)	Total specific activity (U/mg)	Total activity (U/ml)	Soluble protein (mg/ml)	Total specific activity (U/mg)
Ile	ND	1.4	ND	ND	1.7	ND	ND	1.2	ND	ND	1.5	ND
Val	ND	1.7	ND	ND	1.6	ND	ND	1.2	ND	0.005	1.4	0.0036
Leu	0.028	1.2	0.024	0.022	1.4	0.016	ND	1.4	ND	0.043	1.3	0.0331
Phe	ND	0.6	ND	ND	1.3	ND	ND	1.8	ND	ND	1.3	ND
Cys	ND	1.7	ND	ND	1.3	ND	ND	1.3	ND	ND	1.1	ND
Met	0.010	1.8	0.006	0.010	1.2	0.008	ND	1.1	ND	ND	1.4	ND
Ala	0.005	0.8	0.006	0.005	1.8	0.003	ND	1.2	ND	0.009	1.8	0.0050
Gly	ND	1.4	ND	ND	1.5	ND	ND	1.3	ND	ND	1.2	ND
Thr	0.005	1.6	0.003	ND	1.9	ND	ND	1.2	ND	ND	1.2	ND
Ser	0.020	1.4	0.014	ND	1.8	ND	ND	1.8	ND	ND	1.5	ND
Trp	ND	1.8	ND	ND	1.2	ND	ND	1.2	ND	ND	1.3	ND
Tyr	0.037	1.3	0.028	ND	1.3	ND	ND	1.4	ND	0.005	1.2	0.0042
Pro	ND	1.5	ND	ND	1.8	ND	ND	1.1	ND	ND	1.8	ND
His	0.060	0.9	0.067	ND	1.5	ND	0.036	1.6	0.02	ND	1.2	ND
Glu	0.055	1.2	0.046	0.040	1.9	0.021	0.005	1.5	0.00	ND	1.3	ND
Gln	0.050	1.4	0.036	0.060	1.8	0.033	0.012	1.2	0.01	ND	1.2	ND
Asp	0.050	1.3	0.038	0.024	1.4	0.017	0.002	1.4	0.00	ND	1.2	ND

Asn	0.050	1.2	0.042	0.022	1.5	0.015	0.012	1.2	0.01	ND	1.3	ND
Lys	0.012	1.1	0.011	0.042	1.3	0.032	0.001	1.4	0.00	ND	1.1	ND
Arg	0.055	1.5	0.037	0.040	1.5	0.027	ND	1.1	ND	ND	1.5	ND

U/ml of cell-free extract prepared from a 3-ml LB culture in triplicate; ND, not determined

[a]Concentration of soluble proteins means concentration of soluble fraction of crude enzyme solution.

Table S3. INTMSAlign parameters for *Ch*MOX, *At*ADC, *Dm*GDH, *Dm*ODC, *Su*PDH and *Mp*LUC

	*Ch*MOX	*At*ADC	*Dm*GDH	*Dm*ODC	*Me*HNL	*Su*PDH	*Mp*LUC
Types of Blast	Blastp	Blastp	Blastp	Blastp	Blastp	Blastp	Blastp
Database	Non redundant	Non redundant	Non redundant	Non redundant	Non redundant	Non redundant	Non redundant
Sequence of target protein (STP)	*Ch*MOX (GenBank ID; not registered)	*At*ADC (GenBank ID:15227223)	*Dm*GDH (GenBank ID:24649283)	*Dm*ODC (GenBank ID:24586472)	*Me*HNL (GenBank ID: 55469815)	*Su*PDH (GenBank ID: 1842144)	*Mp*LUC (GenBank ID; not registered)
Total number of sequences in the library	5,000	5,000	5,000	5,000	825	158	37
N_{pick}	8	8	8	8	8	8	8
N_{trial}	500	500	500	500	1000	1,000	500
